# A window into extreme longevity; the circulating metabolomic signature of the naked mole-rat, a mammal that shows negligible senescence

**DOI:** 10.1007/s11357-018-0014-2

**Published:** 2018-04-20

**Authors:** Kaitlyn N. Lewis, Nimrod D. Rubinstein, Rochelle Buffenstein

**Affiliations:** Calico Life Sciences LLC, 1170 Veterans Blvd., South San Francisco, 94080 USA

**Keywords:** Metabolomics, Plasma, Amino acid profile, Methionine pathway, Naked mole-rat, Aging, Hibernation, Torpor, Methionine restriction

## Abstract

Mouse-sized naked mole-rats (*Heterocephalus glaber*), unlike other mammals, do not conform to Gompertzian laws of age-related mortality; adults show no age-related change in mortality risk. Moreover, we observe negligible hallmarks of aging with well-maintained physiological and molecular functions, commonly altered with age in other species. We questioned whether naked mole-rats, living an order of magnitude longer than laboratory mice, exhibit different plasma metabolite profiles, which could then highlight novel mechanisms or targets involved in disease and longevity. Using a comprehensive, unbiased metabolomics screen, we observe striking inter-species differences in amino acid, peptide, and lipid metabolites. Low circulating levels of specific amino acids, particularly those linked to the methionine pathway, resemble those observed during the fasting period at late torpor in hibernating ground squirrels and those seen in longer-lived methionine-restricted rats. These data also concur with metabolome reports on long-lived mutant mice, including the Ames dwarf mice and calorically restricted mice, as well as fruit flies, and even show similarities to circulating metabolite differences observed in young human adults when compared to older humans. During evolution, some of these beneficial nutrient/stress response pathways may have been positively selected in the naked mole-rat. These observations suggest that interventions that modify the aging metabolomic profile to a more youthful one may enable people to lead healthier and longer lives.

**Electronic supplementary material:**

The online version of this article (10.1007/s11357-018-0014-2) contains supplementary material, which is available to authorized users.

## Introduction

The comprehensive evaluation of plasma metabolites has the potential to elucidate biomarkers and metabolomic signatures associated with aging (Fuchs et al. 2010; Mishur and Rea 2012; Tomás-Loba et al. 2013; Laye et al. 2015; Hoffman et al. 2016; Wan et al. 2017; Pietzner et al. 2017), lifestyle interventions (dietary restriction/exercise) that prolong good health (De Guzman et al. 2013; Laye et al. 2015; Green et al. 2017; Lewis et al. 2010), and diseased states such as cancer (Nagrath et al. 2011), diabetes (Liu et al. 2013), and cardiovascular disease (Barderas et al. 2011). As such, metabolomic analyses provide a powerful hypothesis-generating tool for mechanisms that may extend life span.

While whole-animal homogenates are used routinely in invertebrate (worm and fly) metabolomics studies (Wan et al. 2017), blood samples are commonly used in vertebrate metabolomics studies (Hoffman et al. 2016; Torell et al. 2017; Pietzner et al. 2017). Since most metabolic pathways operate within cells, the plasma metabolome represents the integration of multiple pathways operating in different tissues, as well as exogenous metabolites including dietary components; metabolites of environmental agents (e.g., pesticides, xenobiotics, and drugs); and microbiome secretions. As such, data interpretation is often challenging; nevertheless, comprehensive, unbiased metabolomic screens have been employed in experimental studies involving traditional model organisms (Kristal et al. 2007; Zhang et al. 2011; Mishur and Rea 2012), as well as exotic animals—such as hibernating ground squirrels (D’Alessandro et al. 2017). This biochemical approach not only provides a useful window into the metabolic pathways involved in response to extreme environmental conditions (e.g., during hibernation) but may also highlight the metabolic signatures pertinent to organisms exhibiting extreme longevity, revealing mechanistic insights or targets that may lead to interventions enabling people to lead healthier and longer lives.

The mouse-sized (35–50 g) naked mole-rat (*Heterocephalus glaber*) is an excellent example of an organism that has naturally achieved both an extended health and life span (Buffenstein 2005, 2008). This endemic inhabitant of the northeastern horn of Africa is the longest-lived rodent known with a maximum life span of at least 32 years (Lewis et al. 2016), exceeding that of the much larger (11 kg) African porcupine by at least 5 years (Hulbert et al. 2007; Buffenstein 2008). This small mammal, which is expected to live 6 years on the basis of size-dependent, allometric assessments, has yet to show the first signs of demographic aging at 12 years. Moreover, well into their third decade, naked mole-rats show no signs of age-associated increased risk of dying, clearly defying Gompertzian laws of mortality (Ruby et al. 2018).

Naked mole-rats not only exhibit exceptional longevity, but they also experience very little decline in physiological and biochemical functions that typically signal advancing age (Buffenstein 2008; Edrey et al. 2011). For example, cardiovascular function is maintained during aging (Grimes et al. 2014). Clearly, unlike other mammals, homeostasis must be maintained in naked mole-rats to abrogate the risk in intrinsic mortality and to protect against environmental challenges that disrupt homeostasis (Lewis et al. 2013). These atypical aging characteristics confirm the status of the naked mole-rat as a model of exceptional biogerontological interest (Buffenstein 2005). Understanding how this rodent species manage to live so much longer than other rodents should provide key, actionable insights into powerful longevity mechanisms.

Naked mole-rats live in large eusocial family groups within a subterranean labyrinth, feeding on underground tubers, bulbs, and roots (Jarvis 1981). Environmental conditions in this belowground milieu of equatorial Africa are thermally stable; atmospheric conditions, given the large number of respiring conspecifics, are likely to be extremely hypoxic, especially in deep underground nests (Sherman et al. 1991). Having inhabited this niche since the early Miocene, naked mole-rats exhibit many ecophysiological adaptations to life below ground (Buffenstein 2000), including pronounced tolerance of hypoxia and anoxia (Park et al. 2017), as well as a low basal metabolic rate and thermolability, with body temperature tracking ambient temperature when individuals are isolated from the colony (Buffenstein and Yahav 1991a).

These adaptations to a subterranean habitat present naked mole-rats with numerous metabolic, biochemical, and molecular challenges. Previous studies have suggested that proteostatic mechanisms (Rodriguez et al. 2014; Pride et al. 2015), cytoprotective (e.g., NRF2) pathways (Lewis et al. 2012, 2015), and concomitant downstream resistance to genotoxic and other stressors, as well as better maintenance of genomic integrity are important contributors to their attenuated aging phenotype (Azpurua et al. 2013; Miyawaki et al. 2016). Together, these pathways may orchestrate a distinct “anti-aging” profile that has a well-defined biochemical signature compared to shorter-lived species, including the well-characterized laboratory (C57Bl/6) mouse. If biochemical signatures are shared with experimentally manipulated (both environmental and genetic) long-lived mouse models, they may elucidate potential mechanisms that broadly extend life in other species.

Determining the mechanisms behind the prolonged life span of the naked mole-rat is daunting, given the limited availability of experimental molecular tools (e.g., antibodies) and genetic approaches, though candidate mechanism for causality could potentially be revealed through careful genomic annotation. In addition, using more global unbiased analyses (e.g. “-omics” platforms) in an experimental design that contrasts the long-lived naked mole-rat with a comparable short-lived mammal may be particularly useful for identifying distinctive characteristics of the naked mole-rat pertinent to attenuation of the aging process. Specifically, a metabolomics profile may reveal, in an unbiased way, metabolites that prove to be biomarkers of age-related survivorship and may ultimately elucidate universal mechanisms involved in slow and successful aging phenotypes. Here, we compare the metabolomic signatures of the naked mole-rat and the shorter-lived, similarly sized, and well-characterized C57Bl/6 mouse. Furthermore, using publically available data, we also compare the circulating metabolome of naked mole-rats to both fasting, metabolically suppressed hibernating squirrels (D’Alessandro et al. 2017), and methionine-restricted rodents (Perrone et al. 2012). We discuss these in the light of data on long-lived Ames dwarf mice (Brown-Borg et al. 2014) and aging humans (Lawton et al. 2008).

## Methods

### Animal maintenance and diet

Nine male naked mole-rats (2–5 years old) and five C57BL6/J male mice (6 months old) were used in this study. The naked mole-rats were part of the well-characterized Buffenstein colony, and the mice were purchased from the Jackson Laboratories (Bar Harbor, Maine, USA) and maintained in the vivarium for at least 1 month prior to use. The ages selected yielded young, healthy individuals that were physiologically age-matched (~ 15–20% of their observed maximum life span). Both species were maintained on a 12-h light-dark cycle. Naked mole-rats were housed in family groups in interconnected systems consisting of tubes and cages of varying sizes to simulate the multi-chambered burrow and tunnel systems that the species inhabits in the wild. Climatic conditions also approximated those found in their native habitat (30 °C; 50% relative humidity), although atmospheric oxygen was ~ 21%. Naked mole-rats met all their nutrient and water needs through an ad libitum supply of fruit and vegetables (bananas, apples, oranges, butternut squash, red bell pepper, romaine lettuce, cucumber, green beans, corn, carrots, and red garnet yams). Although the staple diet of the naked mole-rat (yams) has approximately 10-fold lower levels of total protein than mouse chow (19 g protein/100 g chow; McIsaac et al. 2016; Brown-Borg and Buffenstein 2017), we supplement this with a protein- and vitamin-enriched cereal (Pronutro, Bokomo; 16 g protein/100 g pronutro; www.bokomo.co.za). The naked mole-rat is also coprophagic consuming fecal pellets that contain considerable amounts of the microfauna found in their large cecum (Buffenstein and Yahav 1991b). Although hard to quantify, coprophagic microfaunal intake (approximately 20–24 g protein/100 g microorganisms) (Zubkov et al. 1999), could increase the average protein content of the mole-rat diet to similar levels to that of mice. The mice were group housed (five per cage) at 25 °C and were given ad libitum access to mouse chow (Harlan Teklad 7912) and water. We chose this extensively studied and well-characterized mouse strain in order to ensure that these measurements would be comparable to published findings. These data would then serve as the basis with which to evaluate the naked mole-rat data.

### Experimental procedures

Physiologically age-matched, young male naked mole-rats (*n* = 9) and C57BL6/J mice (*n* = 5) were used in this study. All animals used in this study were sacrificed in the late morning (10.30am -12 noon) after having access to fresh food. Twenty-four hours prior to anesthesia with isoflourane, all animals received an oral 200-μl bolus of sesame oil to serve as control animals for other ongoing studies, and were then sacrificed by cardiac exsanguination. The blood was collected in the presence of EDTA, mixed by inversion and incubated on ice (maximum of 30 min) before centrifugation (5000 g, for 5 min at 4 °C). Plasma samples (~ 0.5–1.5 ml) were collected and stored (− 80 °C) until transfer on dry ice to Metabolon Inc. (Durham, NC, USA), where they were further stored (− 80 °C) without thawing until analysis.

Plasma metabolites were measured by Metabolon Inc. (www.metabolon.com) whose platform and protocol have been described in detail elsewhere (Evans et al. 2009). Briefly, samples as well as a number of standards were analyzed by ultra-high-performance liquid chromatography (based on a Waters ACQUITY UPLC and a Thermo-Finnigan LTQ mass spectrometer) and gas chromatography coupled with tandem mass spectrometry. Compounds were identified by comparing to a chemical reference library including over 1000 standard metabolites to identify the corresponding metabolite and assess quantity. All samples from both naked mole-rats and mice were analyzed together. If concentrations were below detectable limits, a non-detectable value was replaced with the lowest detected value for that metabolite by Metabolon. Unidentified metabolites were given a unique ID for re-analyses at some later time point. The reproducibility of the metabolomics platform used in the current study has been reported previously by Metabolon (Sampson et al. 2013; Moore et al. 2014). Data extraction, metabolite identification, and metabolite quantification were undertaken using proprietary software.

### Metabolomics data and analyses

To enable statistical analysis in samples in which compounds were not detected, the minimum value of that compound that had been detected was imputed (Supplementary Table S1). In order to test whether the abundance of a compound was significantly different between naked mole-rats and mice, we fitted a linear-regression model to the natural log (ln) transformed abundances, setting the mouse as the baseline category, using R-3.4.1. Thus, the slope of this regression, i.e., effect size, is the mean ln(naked mole-rat)/ln(mouse) fold change in metabolite abundance. *P* values quantifying whether this effect was significantly different from zero were adjusted for multiple hypothesis testing using the Benjamini-Hochberg False Discovery Rate (FDR) correction procedure (BH procedure) (Benjamini and Hochberg 1995).

Metabolite abundances were hierarchically clustered using Euclidean distances between the standard-normal transformed ln(abundance) values. The major metabolite clusters were visually determined according the dendrogram topology. To test for enrichment of super- and sub-pathway terms (assigned to each identified metabolite by Metabolon Inc.) in each of the metabolite clusters, we performed a hypergeometric test per each super- and sub-pathway term using the R Bioconductor piano package (Väremo et al. 2013), where *n =* number of identified metabolites in the tested cluster; *k =* number of metabolites assigned to the tested super- or sub-pathway term in the tested cluster; *n =* number of identified metabolites in all clusters; and *K =* number of metabolites assigned to the tested super- or sub-pathway term in all clusters. *P* values were adjusted for multiple hypothesis testing using the BH procedure, and an *α* = 0.05 was used as an adjusted *P* value significance threshold. To test for enrichment of super- and sub-pathway terms in metabolites that are strongly differentially abundant between naked mole-rats and mice (as well as between the late torpor and torpor entrance stages in 13-lined ground squirrels and between the methionine-restricted versus control-fed rats datasets below) we performed an enrichment analysis using the R Bioconductor fgsea package (Sergushichev 2016). Again, *P* values were adjusted for multiple hypothesis testing using the BH procedure, and *α* = 0.05 was used as an FDR-adjusted *P* value significance threshold.

### Comparisons with data from other published studies

The 13-lined ground squirrel metabolomics abundance data were obtained from D’Alessandro et al. (2017), Supplementary Table S2 (data normalized to SA). Only the late torpor (LT) and torpor entrance (Ent) samples were used. We intersected these metabolites by their Kyoto Encyclopedia of Genes and Genomes (KEGG) (Ogata et al. 1999; Kanehisa et al. 2016, 2017) and LIPID MAPS (Sud et al. 2007) accessions with the metabolites profiled in this study by their KEGG and Human Metabolome Database (HMDB) (Wishart et al. 2007, 2009, 2012) accessions. This resulted in 74 metabolites (Supplementary Table S2).

To test for an association between the naked mole-rat versus mouse mean abundance ln(fold change) and the late torpor versus torpor entrance mean abundance ln(fold change) in ground squirrels, we filtered the 74 intersecting metabolites, keeping metabolites with low noise (computed as the coefficient of variation: error/mean of the estimated ln(fold change)) associated with their ln(fold change) estimate since metabolites with noisy ln(fold change) estimates can be randomly distributed around zero and therefore may mask any possible association (Supplementary Fig. S1a). In order to not rely on a specific noise cutoff, we computed the association for the entire range of noise values from both datasets (Supplementary Fig. S1c). If there is no association between the metabolites with strong ln(fold change) in both datasets, we would expect the association between the two to be uniformly low for any given noise cutoff. In contrast, if there was a strong association between the metabolite ln(fold change) in both datasets, we would expect to see a dramatic drop in this association at the noise cutoff, which includes too many noisy, differentially abundant metabolites. This noise cutoff was found at a value of 0.455 (Supplementary Fig. S1c), which resulted in 13 metabolites (Supplementary Table S2).

The mean abundance fold change data of Metabolon Inc. plasma-profiled metabolites in methionine-restricted versus control-fed rats were downloaded from Perrone et al. (Supplementary Table 8 from Perrone et al. 2012). We intersected these metabolites with the metabolites profiled in this study by their Metabolon Inc. compound IDs. This resulted in 213 metabolites (Supplementary Table S3).

We tested to assess if an association existed between the naked mole-rat versus mouse mean abundance ln(fold change) and that of the methionine-restricted versus control-fed rat diet mean abundance ln(fold change) in rats. To do so, we filtered the 213 intersecting metabolites keeping strongly differentially abundant ones; for similar reasons, we applied filtering in the analysis with the 13-lined ground squirrel data (see Supplementary Fig. S1b for the association in the non-filtered data). In this case, however, we did not have the ln(fold change) error estimates and hence kept only metabolites whose fold change FDR-adjusted *P* value was below 0.05, in both datasets, resulting in 63 metabolites (Supplementary Table S3).

## Results and discussion

**Fig. 1:**
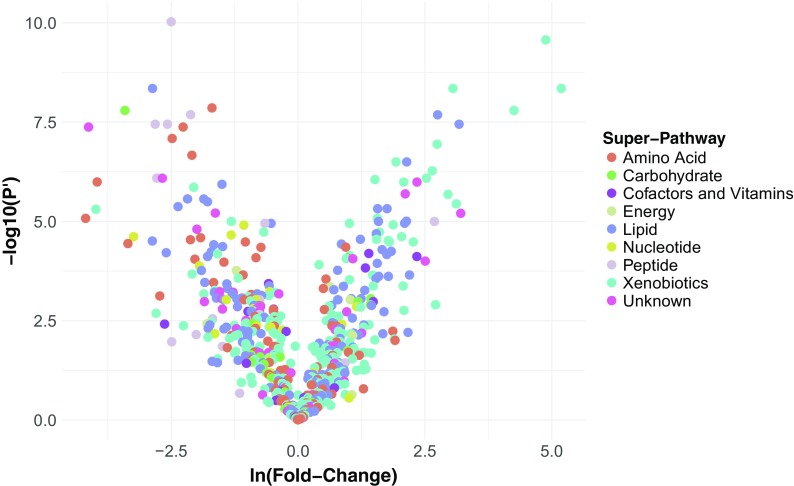
Volcano plot of metabolite abundances in naked mole-rats versus mice. Volcano plot of the statistical significance versus effect size. *X*-axis is mean ln(fold change) (i.e., effect size) in metabolite abundance between naked mole-rats (numerator) and mice (denominator). *Y*-axis is the −log10(FDR adjusted *P* value) (termed *P*′) of the effect. Metabolites are color coded according to the super-pathway they are assigned to by Metabolon, Inc.

In order to identify metabolites whose abundances may be associated with the different life spans of the naked mole-rat versus the C57Bl/6 laboratory mouse, we profiled plasma metabolites from nine adult naked mole-rats and five adult mice using the Metabolon Inc (Durham, NC, USA) platform (see Methods). We obtained abundance measurements for 575 metabolites, 360 of which were identified (Supplementary Table S1). For each of these metabolites, we computed the mean fold change (in natural log space) between the naked mole-rats and mice and its statistical significance. This revealed 370 metabolites with a mean ln(fold change) significantly different from 0 (FDR-adjusted *P* value < 0.05, Fig. 1), among which 238 were identified. Notably, the significantly differentially abundant metabolites were not found to be skewed towards either of the species (*P* value = 0.37; Kolmogorov-Smirnov test), indicating no species-specific systematic biases in our approach.

**Fig. 2:**
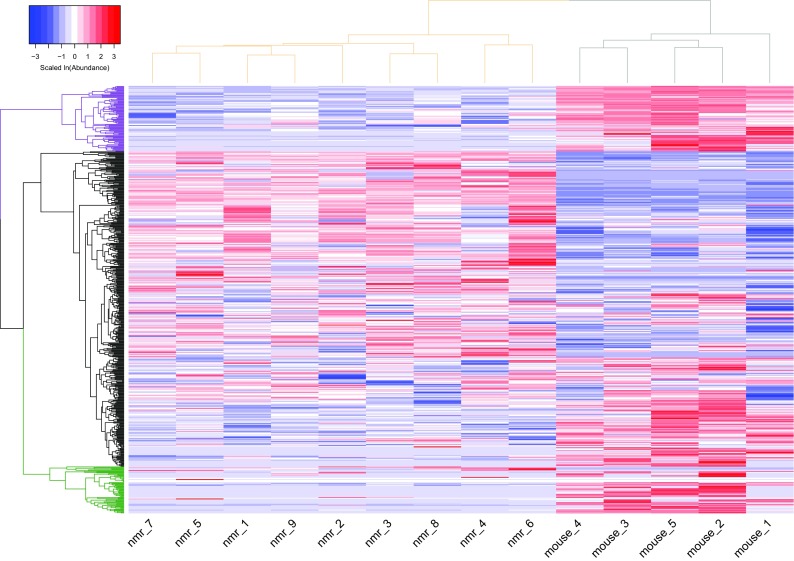
Heat map of naked mole-rat and mouse metabolite abundances. A heat map describing the hierarchical clustering of metabolite abundances from the nine naked mole-rat (nmr) and five mouse plasma samples. ln(abundance) for each metabolite is scaled to have mean of zero and standard deviation of one. The vertical dendrogram is colored according to the two species: naked mole-rat samples are grouped under the beige clade and mouse samples under the gray clade. The horizontal dendrogram is colored according to the three major metabolite clusters separating the naked mole-rat and mouse samples: clades colored purple and green group metabolites downregulated in naked mole-rats relative to mice, where the green clade is enriched in metabolites assigned to the peptides super-pathway and the purple clade is enriched in the lysophospholipid sub-pathway. The black clade groups the metabolites that are upregulated in naked mole-rats relative to mice

**Fig. 3:**
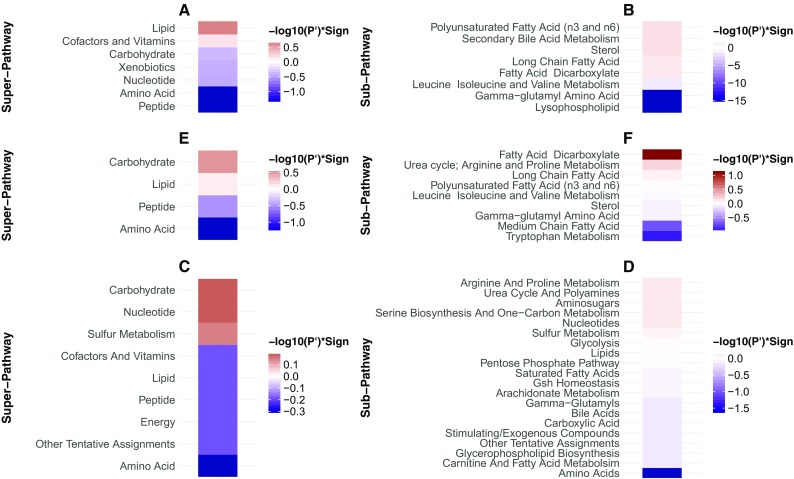
Single-column heatmaps describing the super- and sub-pathway terms that are enriched with metabolites strongly upregulated (red shades) and downregulated (blue shades) in (**A**-**B**) naked mole-rats relative to mice, (**C**-**D**) late-torpor relative to torpor-entrance in 13-lined ground squirrels, and (**E**-**F**) methionine-restricted relative to control-fed rats. Colors correspond to the -log10(FDR adjusted P-value) (termed P’) of the enrichment set analysis statistical test (see Methods) multiplied by the sign of the mean ln(fold-change) of metabolite abundance

We carried out hierarchical clustering of the metabolite abundances across all samples; the naked mole-rat and mouse samples grouped separately, and we found three main abundance clusters, two of which were downregulated in naked mole-rats relative to mice (Fig. 2). Searches for significantly enriched super- and sub-pathway terms with which each of these three metabolite clusters are assigned (Supplementary Table S4) revealed in one of the naked mole-rat downregulated clusters (marked by the green clade in Fig. 2) a significant enrichment in the peptides super-pathway (FDR-adjusted *P* value = 1.16 × 10^−4^) and in the fibrinogen cleavage sub-pathway nested within the peptide super-pathway (FDR-adjusted *P* value = 0.027). In addition, a significant enrichment of the lysophospholipid sub-pathway (FDR-adjusted *P* value = 3.12 × 10^−11^) was found in the other naked mole-rat downregulated cluster (marked by the purple clade in Fig. 2). We further performed a super- and sub-pathway enrichment set analyses among the most strongly differentially abundant metabolites (Supplementary Table S4). This revealed that metabolites that are strongly downregulated in naked mole-rat relative to mouse are significantly enriched in the peptides and amino acid super-pathways (both FDR-adjusted *P* values = 0.04) (Fig. 3a), as well as an amino acid sub-pathway (leucine, isoleucine, and valine metabolism, FDR-adjusted *P* value = 0.04); a peptide sub-pathway (gamma-glutamyl amino acid, FDR-adjusted *P* value < 10^−16^); and a lipid sub-pathway (lysophospholipid, FDR-adjusted *P* value < 10^−16^). It also revealed that metabolites that are strongly upregulated in naked mole-rat relative to mouse are significantly enriched in several lipid sub-pathways involved in membrane composition, sterols, and secondary bile metabolism (all FDR-adjusted *P* values = 0.04) (Fig. 3b).

### Metabolites with species differences in the amino acid super-pathway

Within the amino acid super-pathway, the most conspicuous species differences were in circulating levels of glycine, methionine, leucine, and tyrosine, which were 17–50% lower in naked mole-rats than in mice (all FDR-adjusted *P* values < 8.9 × 10^−4^). Similarly, the long-lived insulin receptor substrate 1 null (Irs^−/−^) mice also have very low circulating levels of methionine. In contrast, long-lived Ames dwarf mice reportedly have increased plasma methionine levels relative to control mice, though levels of other amino acid metabolites were more in keeping with the naked mole-rat. Tryptophan levels in naked mole-rats were also ~ 55% those of mice (FDR-adjusted *P* value = 0.01), and naked mole-rat levels of serotonin, of which tryptophan is the precursor, were very low (~ 6.5%) relative to those in mice (FDR-adjusted *P* value = 7.5 × 10^−4^). In contrast, levels of degradation products of tryptophan were all higher in naked mole-rats than in mice (kynurenine, indoleacetate, N-acetylkynurenine, all FDR-adjusted *P* values < 0.46). Similar to tyrosine, the levels of the aromatic amino acid phenylalanine were also lower in naked mole-rats compared to mice (~ 61%; FDR-adjusted *P* value = 0.0016), while again the levels of many of phenylalanine’s degradation products (e.g., phenylacetate, phenylacetylglycine, and phenylacetylglutamine) were higher in naked mole-rats (all FDR-adjusted *P* values < 0.36). These data suggest that when compared to mice, naked mole-rats show greater levels of amino acid degradation. Notably, circulating levels of both branched-chain amino acids (leucine, isoleucine, and valine) and aromatic amino acids have been positively correlated with insulin resistance and implicated in diabetes (Wang et al. 2011). Naked mole-rats exhibit exquisite sensitivity to insulin tolerance tests (Kramer and Buffenstein 2004) and naturally also display low-fasting blood glucose levels.

Although naked mole-rats are fed a low-protein diet of fresh fruits and vegetables, with protein level 10-fold lower than the mouse diet (McIsaac et al. 2016), this seems an unlikely explanation for the naked mole-rats’ lower amino acid levels, since their diet is supplemented with a high-protein cereal mix (Pronutro 16 g protein/100 g) and with unmeasurable levels of proteins from the microbiome-rich fecal matter digested during coprophagy. Moreover, the low-circulating proteome cannot be attributed to a prolonged fasting state in the naked mole-rat for these rodents tend to eat as soon as fresh food is provided which in this case was 2–3 h before euthanasia. As such, they are likely to have fed more recently than the mice in the study that generally consume the bulk of their food during the scotophase. Notably, a decline in amino acid abundance was also found to be a hallmark of dietary restriction in flies, in almost every tissue and age from which metabolites were profiled (Laye et al. 2015). Additionally, low-circulating branched-chain and aromatic amino acids may also reflect the relationship of these amino acids with insulin sensitivity and glucose metabolism. However, the metabolite profiles of long-lived *Caenorhabditis elegans* mutants (namely *daf-2* insulin/IGF-1-receptor mutants) show increases in pools of branched-chain amino acids compared to wild-type worms (Fuchs et al. 2010) and these levels declined considerably in old worms (Davies et al. 2015). These inconsistencies between studies are difficult to reconcile but could be due to the considerable differences in the profiled tissues (plasma in our study and whole worm homogenates (Fuchs et al. 2010)) and evolutionary divergence between these species.

In contrast to these downregulated amino acids in naked mole-rats, the levels of glutamate were significantly higher in naked mole-rats (~ 1.9-fold; FDR-adjusted *P* value = 0.01). Glutamate is functionally critical in glutathione (GSH) synthesis, an important signaling molecule and also a major excitatory neurotransmitter, and its product, GABA, as a key inhibitory neurotransmitter (Brosnan and Brosnan 2013). Conspicuously, gamma-glutamyl amino acid (GGAA) levels were significantly lower in naked mole-rats relative to mice, (e.g., gamma-glutamylleucine, gamma-glutamylvaline, gamma-glutamyltyrosine, at 13–16% of the mouse levels, all FDR-adjusted *P* values < 8.1 × 10^−7^). GGAA synthesis is catalyzed by gamma-glutamyltranspeptidase (GGT) and involves the transfer of the glutathione (GSH) glutamyl moiety to free amino acids to form GGAAs. These are then transported across the cell membrane and converted by intracellular gamma-glutamylcyclotransferase to the corresponding amino acids and 5-oxoproline. This process is dependent upon the availability of GSH (Griffith et al. 1979). GSH levels were not detected in this metabolomic screen and may reflect the low levels previously reported in naked mole-rats (Andziak et al. 2006). The 6.7-fold higher levels of glutamate may be linked to the low levels of GSH, for glutamate is needed in the first steps of GSH synthesis.

Another dramatic species difference in the amino acid super pathway was found to be in the levels of creatine, which in naked mole-rats was less than half of that observed in mice (FDR-adjusted *P* value = 0.0025). Such low levels likely reflect the naked mole-rat’s strict herbivorous diet and concomitant low creatine levels in the fruit and vegetables it consumes. Low levels of this organic acid may also reflect the lower basal metabolic rate of the naked mole-rat, 66–75% of that of mice (Buffenstein and Yahav 1991b). Creatine plays a key role in ATP recycling and in ensuring a continuous energy supply, and its levels decrease with reduced energy demands. Creatinine, the degradation product of creatine excreted through the urine, was present at significantly higher levels in naked mole-rats relative to mice (Supplementary Table S1). In the absence of dehydration, circulating creatinine concentration is often used as an indicator of glomerular filtration rate or renal function rather than an indicator of ATP flux. These pronounced creatinine-species differences may thus be indicative of more efficient removal of waste products in the longer-lived species.

The significantly lower levels of circulating amino acids and peptides and higher levels of degradation products in naked mole-rats relative to mice may be indicative of the high levels of proteolytic activity, rapidly degrading proteins into small peptide chains. Both liver lysate and cell culture data indicate that naked mole-rats have higher rates of proteasomal activity and autophagy than mice (Rodriguez et al. 2014; Pride et al. 2015) and suggest that amino acid recycling in tissues may contribute to sustained and well-maintained proteostasis.

### Metabolites with species differences in the lipid super-pathway

In addition to the amino acid and peptide super-pathways, the lipid super-pathway was found to be enriched in metabolites with a strong species difference; in many cases, with higher levels in naked mole-rat sub-pathways relative to mice. These included several fatty acid metabolites as well as components of bile metabolism. A similar, albeit-graded enrichment of lipid-associated metabolites was observed in the livers of young mice subjected to different degrees of caloric restriction where several lipid metabolites were increasingly upregulated with increasing severity of caloric restriction (Green et al. 2017). Similarly, circulating metabolites from both young mice fed ad libitum and aged mice maintained on 40% caloric restriction when both compared with that of aged mice maintained with ad libitum access to food (De Guzman et al. 2013) also exhibited upregulated lipid super-pathways; Specifically, calorically restricted 26- and 3-month-old C57Bl/6 mice showed similar abundances of sphingomyelins, sterols, lysophosphatidylcholines, and membrane phospholipids. Although in the present study, the young healthy mice were slightly older than 3 months (6 months old), naked mole-rats showed 15.6- and 8.45-fold higher (both FDR-adjusted *P* values < 7.69 × 10^−7^) palmitoyl sphingomyelin (d18:1/16:0) and stearoyl sphingomyelin (d18:1/18:0) levels, respectively, compared to mice. In addition, most of the identified medium- and long-chain fatty acids, as well as sterols, were also found to have significantly higher levels in the naked mole-rats relative to mice; notable exceptions were nonadecanoate (19:0), docosahexaenoate (DHA; 22:6n3), as well as the essential fatty acid linoleate (18:2n6), which were lower in naked mole-rats relative to mice. Linoleate is commonly converted into arachidonate and other PUFAs that are elevated in the naked mole-rats. The increase in PUFAs was associated with a significant drop in lysophospholipids and an increase in sphingolipid metabolism.

Differences in saturated and polyunsaturated fatty acids reflect, in part, the different membrane phospholipid compositions in peripheral tissues (Hulbert et al. 2006). The susceptibility of membranes to oxidative stress depends in large part on the proportion of n-3 and n-6 PUFAs in the cell membrane phospholipid; membrane phospholipids have a lower peroxidation index if there is proportionately less of the n-3 PUFA docosahexanoic acid and high levels of the n-6 PUFA arachidonic acid (Hulbert et al. 2007). Naked mole-rats have significantly more n-6 phospholipids. Membrane phospholipids are constantly being degraded as part of membrane repair and in the process, lysophospholipids are formed by lipolytic enzymes. Lysophospholipids are biologically active molecules involved in numerous physiological and pathological processes, such as inflammation, apoptosis, carcinogenesis, angiogenesis, and regulation of metabolic diseases (Kim et al. 2014). Their levels increase on high fat diets and during disease progression (Suárez-García et al. 2017). As such, the low levels of plasma lysophospholipids (enrichment test FDR-adjusted *P* value < 10^−16^) in the naked mole-rats may reflect their disparate membrane composition and greater membrane stability (Hulbert et al. 2006) and lower fat diet than mice. Although highly speculative, this lysophospholipid profile may contribute to (or reflect) their generally better health than mice and their greater resistance to age-associated diseases.

In contrast to lysophospholipids, two other membrane lipids ~ sphingolipid metabolites were markedly higher in the naked mole-rat than in the mouse: palmitoyl sphingomyelin (d18:1/16:0) and stearoyl sphingomyelin (d18:1/18:0) were, respectively, 15- and 11-fold higher levels in naked mole-rats (both FDR-adjusted *P* values < 7.6 × 10^−7^). Sphingomyelins are synthesized from phosphorylcholine, sphingosine, and an acylated group, such as a fatty acid. Palmitoyl sphingomyelin is a form of sphingomyelin containing palmitate (16:0) at the variable acylation position. Palmitoyl sphingomyelin interacts with cholesterol in ordered lipid rafts in cell membranes, whereas stearoyl sphingomyelin is also a major constituent of membranes interacting with cholesterol and is a key component of vesicular trafficking (Gault et al. 2010). There is also some evidence that through these interactions, sphingomyelin protects cholesterol from oxidation (Patzer and Wagner 1978). In contrast, the third metabolite identified in this sub-pathway, sphingosine, the sphingoid base backbone of the two other sphingolipids, was found to be present at significantly lower levels than in mice (FDR-adjusted *P* value = 0.0013). Amphipathic sphingolipids are not only a major constituent of cell membranes, in particular the myelin sheath of nerves, but are also a major constituent of biologically active molecules controlling many cellular processes, including cell division, differentiation, apoptosis, and signaling (Milhas et al. 2010; Pralhada Rao et al. 2013). Dysregulation of sphingolipid metabolism is linked to high levels of sphingomyelinase activity, sphingomyelin degradation, and increased ceramide formation. As such, pathologically low levels of spingomyelins are linked to ER stress, inflammation, and insulin resistance, contributing to proliferative disorders (e.g., hepatocellular carcinoma), metabolic disorders (type 2 diabetes mellitus), as well as neuronal disorders such including Alzheimer’s, amyotrophic lateral sclerosis, and Parkinson’s disease (Straczkowski et al. 2004; Pralhada Rao et al. 2013).

Species differences in fatty acid abundances also likely reflect the greater reliance of naked mole-rats on microbial fermentation products, most notably short-chain fatty acids, produced by a myriad of microorganisms in their large cecal vat (Buffenstein and Yahav 1991b). All detected circulating sterols, both plant- and animal-based, were found to have significantly higher levels in naked mole-rats relative to mice (Supplementary Table S1; all FDR-adjusted *P* values < 0.02). As an example, cholesterol levels were ~ 2.5-fold higher in naked mole-rats. Although cholesterol levels are strongly positively associated with cardiovascular disease in humans (Cholesterol Treatment Trialists’ (CTT) Collaboration 2010), it is a precursor for all steroid hormones, of which testosterone and cortisol showed higher levels in naked mole-rats relative to mice (Supplementary Table S1; FDR-adjusted *P* values = 0.1 and 0.03, respectively). In contrast, naked mole-rats showed significantly lower plasma levels of corticosterone (Supplementary Table S1; FDR-adjusted *P* value = 8.4 × 10^−4^) and higher levels of cortisol (Supplementary Table S1; FDR-adjusted *P* value = 0.02) than in mice. This confirms previous observations using ELISA and RIA assays (Buffenstein and Pinto 2009), which showed that naked mole-rats, like humans, use cortisol as their main glucocorticoid in contrast to mice, which use corticosterone. The biological significance of this difference in glucocorticoids remains poorly understood.

Bile salts are also derived from cholesterol and play key roles in emulsifying lipid aggregates and in facilitating transport of these hydrophobic lipids in the predominantly hydrophilic plasma. Large species differences in circulating levels of bile salts were also evident, with the exception of 12-dehydrocholate and tauroursodeoxycholate. Three of these bile salts had close to an order of magnitude or higher concentrations in naked mole-rats relative to mice, namely deoxycholate (9-fold, FDR-adjusted *P* value = 2.2 × 10^−4^); glycodeoxycholate (24-fold; FDR-adjusted *P* value = 3.56 × 10^−8^); and ursodeoxycholate (8.7-fold; FDR-adjusted *P* value = 6.2 × 10^−3^). These bile salts are primarily formed through microbial metabolism of cholic acid although ursodeoxycholate may also be formed in the liver. Such high levels of secondary bile metabolites are usually associated with a diet low in fiber and high in fat; this certainly is not the case for the naked mole-rat.

High levels of bile salts, in particular those of secondary bile metabolism (e.g., deoxycholate and glycodeoxycholate) in the serum reportedly are indicative of liver disease. They are often considered both cytotoxic and carcinogenic, thereby implicated in the pathogenesis of gastrointestinal neoplasia (Centuori and Martinez 2014). This is attributed to the hyperactivation of the EGFR-MAPK pathway and the concomitant activation of the AP-1 proto-oncogene and suppression of the p53 tumor-suppressor gene. These hydrophobic bile acids are also known to induce ER stress (Payne et al. 2005), oxidative stress (Washo-Stultz et al. 2002; Sokol et al. 2005; Jenkins et al. 2007; Payne et al. 2007), mitochondrial stress (Washo-Stultz et al. 2002; Rolo 2004; Sokol et al. 2005; Payne et al. 2005, 2007), and DNA damage (Glinghammar 2002; Bernstein et al. 2006; Jenkins et al. 2007; Rosignoli et al. 2008). Naked mole-rats seem to have once again circumvented toxic consequences associated with high levels of secondary bile metabolites, showing surprisingly high levels of resilience reminiscent of their cells’ high levels of resistance to cytotoxin exposure in vitro (Lewis et al. 2012).

In contrast to deoxycholate, ursodeoxycholate is known to have chemopreventative anti-tumorigenic properties, although its mechanism of action negatively regulating pathways activated by deoxycholate remains poorly understood (Centuori and Martinez 2014). Both rat and humans treated with this compound have shown significant reductions in both tumor development and ulcerative cholitis (Earnest et al. 1994; Tung et al. 2001; Pardi et al. 2003; Alberts et al. 2005).

Notwithstanding the identity of many significantly differentially abundant metabolites, some with staggering fold changes cannot be currently identified (e.g., X - 16947_1 being ~ 180 times more abundant in naked mole-rats than in mice; FDR-adjusted *P* value = 4.5 × 10^−9^). While it is conceivable that these unidentified metabolites may reflect novel mechanisms linked to species longevity, the possibility that they are related to species differences in the microfauna and flora or environmental substances cannot be excluded. Even if so, these differences in microbial secretions and xenobiotics may also contribute to the sustained good health and exceptional longevity of the naked mole-rat.

### Comparisons with data from other published studies

Hibernation is generally believed to be an energy-saving adaptation that allows endotherms to reside year-round in highly seasonal climates (Boyer and Barnes 1999). Although naked mole-rats do not employ torpor on a seasonal or daily basis, they share many features with torpid mammals; for example, metabolic rate, thyroid hormone levels, and body temperature are all lower than that commonly observed in euthermic mice and rats. A recent study performed a comprehensive analysis of metabolite concentrations in 13-lined ground squirrels during the various torpor and awake states (D’Alessandro et al. 2017). Contrasting metabolite abundances between late torpor and entrance to torpor (awake/euthermic), the authors reported several metabolites involved in the amino acid super-pathway to have significantly different abundances between these two torpor states (Supplementary Table S4 and Fig. 3c, d) (D’Alessandro et al. 2017). During their late torpor, ground squirrels do not eat and mainly rely on lipids supplemented with protein catabolism to fuel their energetic demands. We thus hypothesized that the amino acid catabolic state in late torpor versus torpor entrance in 13-lined ground squirrels resembles that of the naked mole-rat metabolome and mimics calorically restricted or methionine-restricted rodents and long-lived mice.

**Fig. 4:**
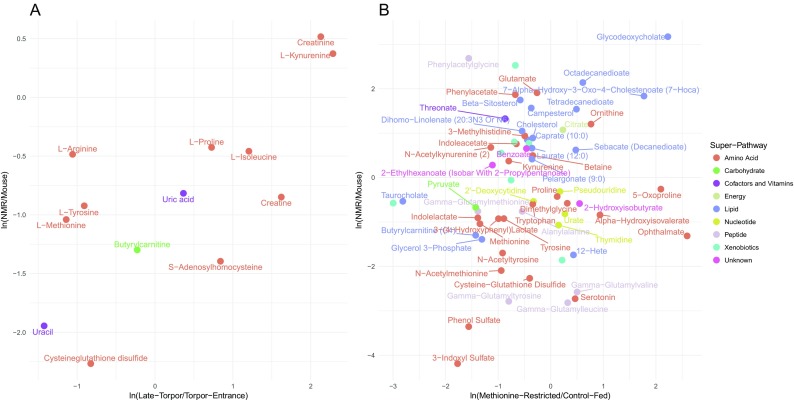
Association between metabolite fold changes in naked mole-rats relative to mice and other compared studies. Mean ln(fold change) of metabolite abundances in naked mole-rats (NMR) versus mice versus: **a** late torpor versus torpor entrance in 13-lined ground squirrel and **b** methionine-restricted versus control-fed rats. Metabolites selected for these analyses are described in the “Methods” section. Metabolites are color-coded according to the super-pathway they are assigned to by Metabolon, Inc.

To test the former hypothesis, we quantified the association between the metabolite abundance fold changes in naked mole-rats versus mice to that in late torpor versus torpor entrance in 13-lined ground squirrels (see “Methods”). This revealed a strong positive association (Pearson correlation coefficient = 0.7; *P* value = 7 × 10^−3^) (Fig. 4a), mostly driven by metabolites in the amino acid super-pathway such as high fold changes in creatinine levels and low fold changes in L-methionine levels, indicative of ongoing amino acid catabolism in both naked mole-rats relative to mice and later-torpor relative to early torpor states in 13-lined ground squirrels. Other metabolites, such as L-proline, L-isoleucine, and S-adenosylhomocysteine did not show this general trend (Fig. 4a).

Similar to the 13-lined ground squirrel study, a metabolomics study contrasting rats on a methionine-restricted diet with control-fed rats reported significant differential abundances in metabolites in the amino acid, peptide, and lipid super-pathways (Perrone et al. 2012) (Supplementary Table S4 and Fig. 3e, f). Quantifying the association between the metabolite abundance fold changes in naked mole-rats versus mice to that in methionine-restricted versus control-fed rats additionally revealed a positive association (Pearson correlation coefficient = 0.17; *P* value = 0.17) (Fig. 4b), which becomes statistically significant after removal of five outliers (taurocholate, ophthalmate, 5-oxoproline, phenylacetylglycine, and X – 02029; Pearson correlation coefficient = 0.3; *P* value = 0.02), hence additionally highlighting shared traits that may be directly pertinent to extreme longevity. This positive relationship is mainly driven by downregulated metabolites in the amino acid super-pathway and upregulated metabolites in the lipid super-pathway, in both studies. For example, both methionine-restricted rats and naked mole-rats show low circulating levels of pyruvate, succinate, and fumarate relative to control-fed rats and mice, respectively, in contrast to citrate which shows the opposite trend. These differences may reflect the lower metabolic rate and concomitant declines in cellular respiration.

## Conclusion

**Fig. 5:**
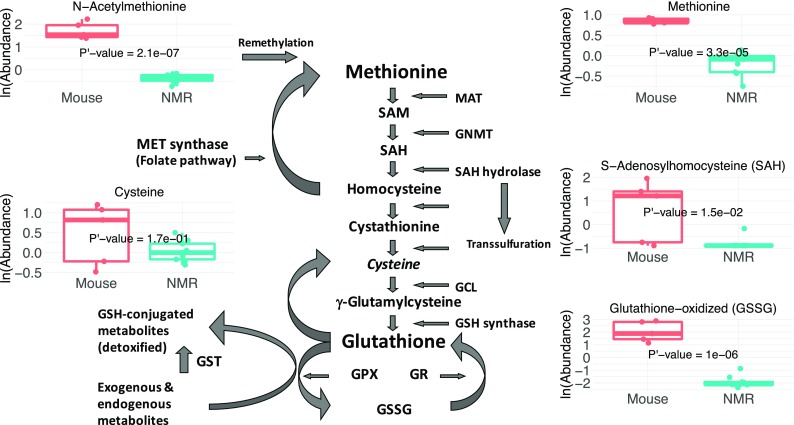
The methionine metabolism pathway. Schematic diagram of the methionine metabolism pathway in the center with the naked mole-rat (NMR) and mouse boxplots of five key metabolites of this pathway, showing the downregulation in naked mole-rats relative to mice

Using metabolomic screens, we observed that young, healthy naked mole-rats and mice exhibit markedly distinct plasma metabolite profiles, with the most pronounced differences evident among circulating amino acid, peptide, and lipid metabolites. Moreover, the largest differences in metabolite levels were observed in several as-yet unidentified metabolites. Although the biological significance of these differences in circulating metabolites is difficult to discern, the differential findings appear to align with similar trends observed in several rodent models of extended longevity. Conspicuous differences observed between naked mole-rats and mice are evident in the methionine pathway, with plasma levels of methionine 2.8-fold greater in mice than in naked mole-rats and similar low levels of downstream metabolites of methionine metabolism (Fig. 5). The Orentreich group has shown unequivocally that both rats and mice maintained on a methionine-restricted diet live significantly longer than those fed normal chow (Orentreich et al. 1993; Zimmerman et al. 2003; Miller et al. 2005). Moreover, these methionine-restricted rodents not only show greater activity but also prolonged good health with both a lower incidence and delay in age-onset of cancer, cataracts, inflammation, and insulin insensitivity (Miller et al. 2005; Stone et al. 2014; Sinha et al. 2014). Although the low methionine plant-based naked mole-rat diet is supplemented with both a protein-rich cereal and the high protein content of digested microbiota in fecal samples, naked mole-rats have low serum levels of methionine and other components of this pathway and, similar to dwarf mice (albeit profiled from liver tissue and not plasma), share many common traits with methionine-restricted mice including resistance to cancer as well as enhanced stress resistance (Brown-Borg et al. 1996; Buffenstein 2005). It is possible that these shared traits of similar perturbations in circulating methionine metabolites reflect that methionine metabolism is an integral modulator of both health and life span (Uthus and Brown-Borg 2006; Brown-Borg and Buffenstein 2017).

These metabolite abundance differences observed between naked mole-rats and mice were found to resemble that of hibernating ground squirrels and rats subjected to dietary/amino acid restriction. All these rodents possess several traits in common such as lower body temperatures, low thyroid metabolism, low insulin levels, and reduced metabolic rates (Buffenstein et al. 2001). These findings suggest a possible link between the downstream effects of a lower metabolic rate, altered protein metabolism, and increased life span. In addition, the significantly lower levels of specific amino acids, most notably, methionine, serine, aspargine, and aminoadipate, and energy metabolites (hydroxybutyrate, diphosphate, nicotinamide) in hibernators (D’Alessandro et al. 2017) are correlated with body temperature during torpor and may also contribute to the lower body temperature and thermal lability of both dwarf mice (Hunter et al. 1999; Gesing et al. 2012) and naked mole-rats (Buffenstein and Yahav 1991a). Both hibernating squirrels and dwarf mice also exhibit signs of increased cytoprotection. NRF2 protein levels are constitutively increased not only in the naked mole-rat, but NRF2-signaling levels are also increased in dwarf mice (compared to wild-type littermates) and hibernating squirrels (compared to fully aroused squirrels) (Pier Jr et al. 2008). This mechanism is hypothesized to prevent the accumulation of oxidative damage that can potentially contribute to disease states including cancer and rapid aging.

With respect to humans, a study looking at the effect of age on metabolite levels reported that in young adults, the levels of essential and nonessential amino acids, urea, ornithine, polyamines, and oxidative stress markers (e.g., hippurate) are lower than in older adults (Lawton et al. 2008). These low metabolite levels are supportive of the youthful naked mole-rat metabolomic profile when compared to mice. Albeit, given the variability in human phenotypes due to many uncontrolled environmental effects, as well as co-factors such as race, sex, body mass index (BMI), and health status, this analogy warrants confirmation from larger and better-controlled studies.

In summary, we report here that the unbiased and comprehensive metabolomic analysis of differences in abundances of circulating metabolites between naked mole-rats and mice surprisingly reveals commonalities in amino acid profiles with both hibernating and methionine-restricted mammals. These data also concur with findings from Ames dwarf mice and caloric restricted mice and human studies in which people with a youthful phenotype have lower levels of amino acids, creatine, and tricarboxylic-acid metabolites than older people. Collectively, we speculate that low circulating levels of amino acids may be a metabolic signature in organisms that exhibit prolonged longevity. It is important to emphasize that these are still ongoing studies with many key metabolites currently unidentified. As we progress and begin to identify unknown metabolites, it is likely that additional novel mechanisms that contribute to the exceptional longevity of naked mole-rats will be elucidated. Finally, the observed similarities between experimentally manipulated long-lived mice and the naked mole-rat, including increased body fat, increased cellular stress resistance, increased cancer resistance, and now similar metabolomic signatures supports the possibility of convergent mechanisms contributing to prolonged life span.

## Electronic supplementary material

Supplementary Fig. S1 (PDF 8 kb)

Supplementary Table S1 (CSV 205 kb)

Supplementary Table S2 (CSV 84 kb)

Supplementary Table S3 (CSV 18 kb)

Supplementary Table S4 (CSV 7 kb)
