# Supplementary figures and images for: A window into extreme longevity; the circulating metabolomic signature of the naked mole-rat, a mammal that shows negligible senescence
Source: GeroScience. 2018 Apr 20;40(2):105–21. doi: 10.1007/s11357-018-0014-2 (PMC5964061; doi:10.1007/s11357-018-0014-2)

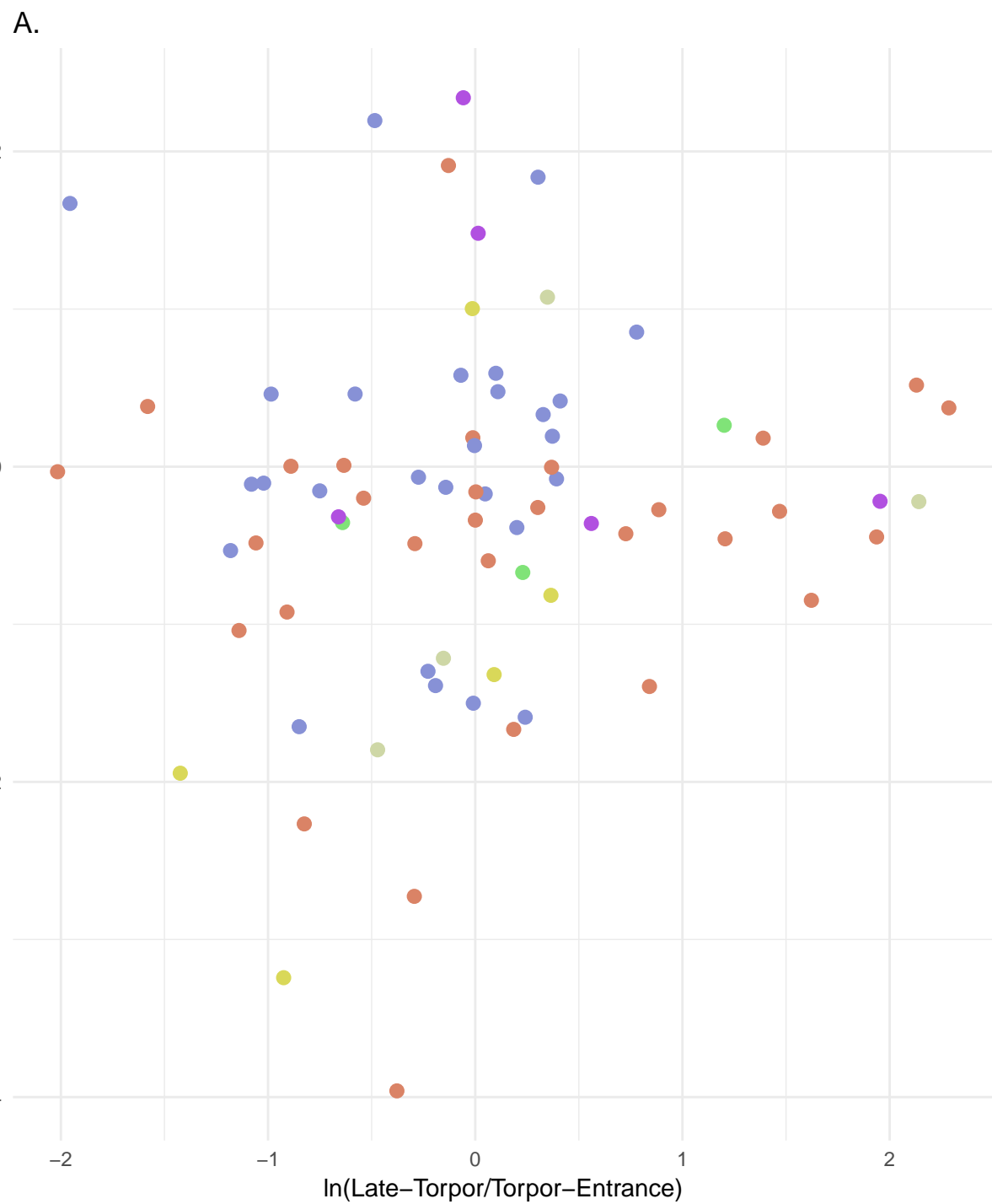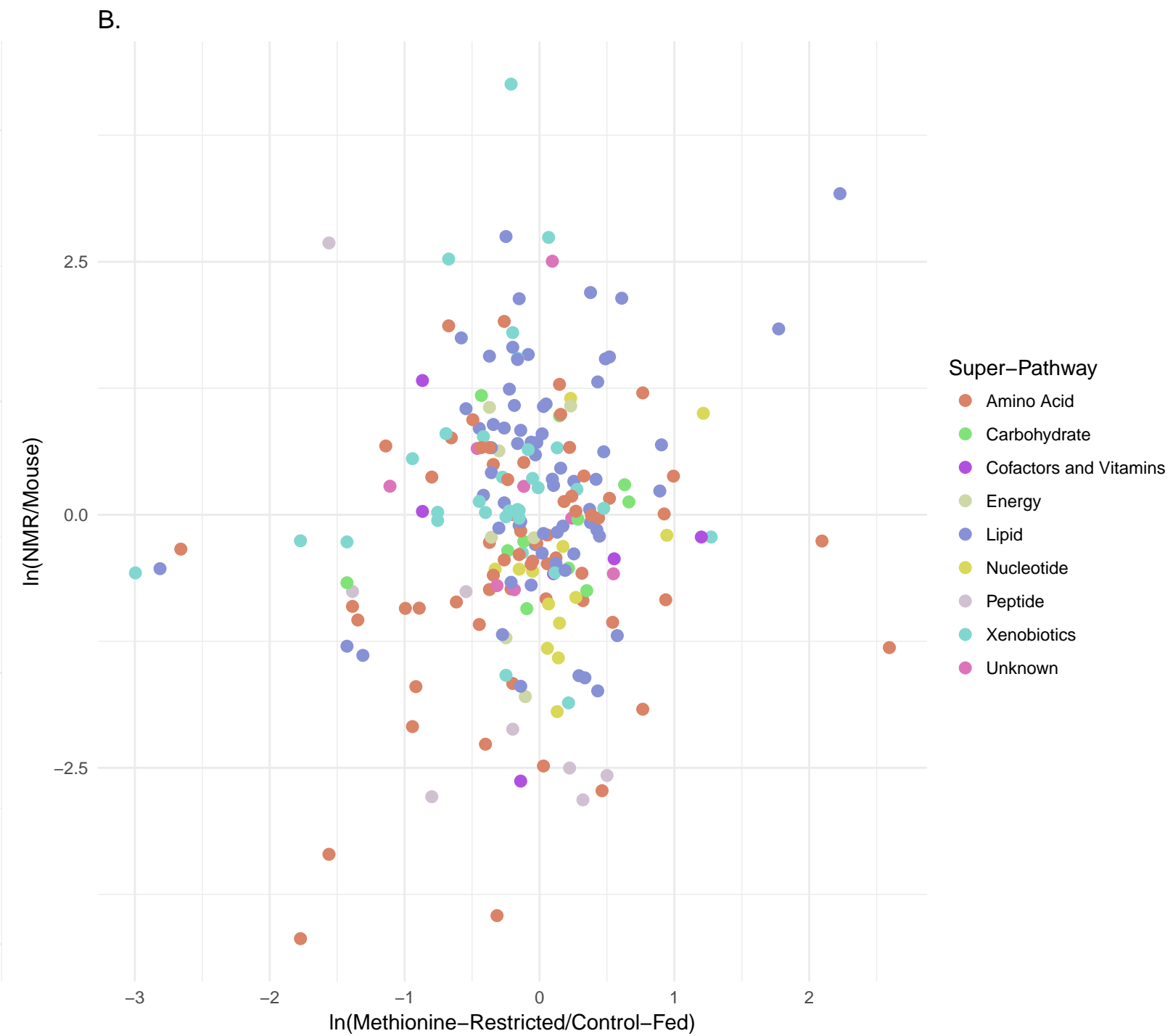

Supplement: Supplementary file 1 — (PDF 8 kb) [file 11357_2018_14_MOESM1_ESM.pdf]
